# Neurological and Cardiovascular Complications Revealing Biermer's Disease: A Case Report

**DOI:** 10.7759/cureus.58601

**Published:** 2024-04-19

**Authors:** Fouad Haddad, Zineb Boukhal, Fatima Zahra El Rhaoussi, Mohamed Tahiri, Wafaa Hliwa, Ahmed Bellabah, Badre Wafaa

**Affiliations:** 1 Gastroenterology and Hepatology, Ibn Rochd University Hospital Center, Casablanca, MAR; 2 Faculty of Medicine and Pharmacy, Hassan II University, Casablanca, MAR

**Keywords:** vitamin b12 supplementation, secondary cardiac dysautonomia, deep vein thrombosis (dvt), neurological disorder, anti-intrinsic factor antibodies, fundic atrophy, vitamin b12, macrocytosis, pernicious anemia

## Abstract

Biermer's disease (BD) or pernicious anemia (PA) is an autoimmune atrophic gastritis characterized by the absence of intrinsic factor (IF) secretion, leading to malabsorption of vitamin B12 in the ileum. Its clinical manifestations are primarily hematological, with neuropsychiatric and cardiovascular manifestations being less common. We present the case of a patient with PA diagnosed based on neurological and cardiovascular complications.

The patient, a 56-year-old man with no specific medical history, presented with an episode of melena without other associated digestive symptoms. He also complained of memory and gait disturbances. Clinical examination revealed a cerebellar ataxia with impaired proprioceptive and vibratory sensitivity, and a swollen and red right lower limb with a positive Homan sign. The blood count showed macrocytic anemia. Gastroscopy revealed flattened fundic folds resembling a fundus appearance, and histopathological examination confirmed fundic atrophic gastritis with pseudopyloric metaplasia and lymphoplasmacytic infiltration. Anti-intrinsic factor antibodies were positive, while anti-parietal cell antibodies were negative. Vitamin B12 levels were severely low, and vitamin B9 levels were normal. TSH and HbA1c levels were within normal ranges. The abdominal CT scan showed no abnormalities. Lower limb Doppler ultrasound confirmed the diagnosis of deep vein thrombosis (DVT). Cardiac evaluation revealed sinus bradycardia suggestive of secondary dysautonomia. Therapeutically, the patient was started on vitamin B12 supplementation and anticoagulant therapy for DVT, resulting in a good clinical and biological outcome.

## Introduction

Pernicious anemia (PA) is an autoimmune disease, and it accounts for 20-50% of adult-onset vitamin B12 deficiencies. It is linked to a lack of intrinsic factor (IF) secretion secondary to atrophic gastritis and the presence of antibodies directed against IF [1]. Typically, it leads to hematological abnormalities and digestive disorders. Neuropsychiatric symptoms are varied and of varying severity. Additionally, PA is considered a risk factor for cardiovascular diseases associated with atherosclerosis secondary to hyperhomocysteinemia [2]. We discuss a case of PA revealed by neurological and cardiovascular complications.

## Case presentation

**Figure 1:**
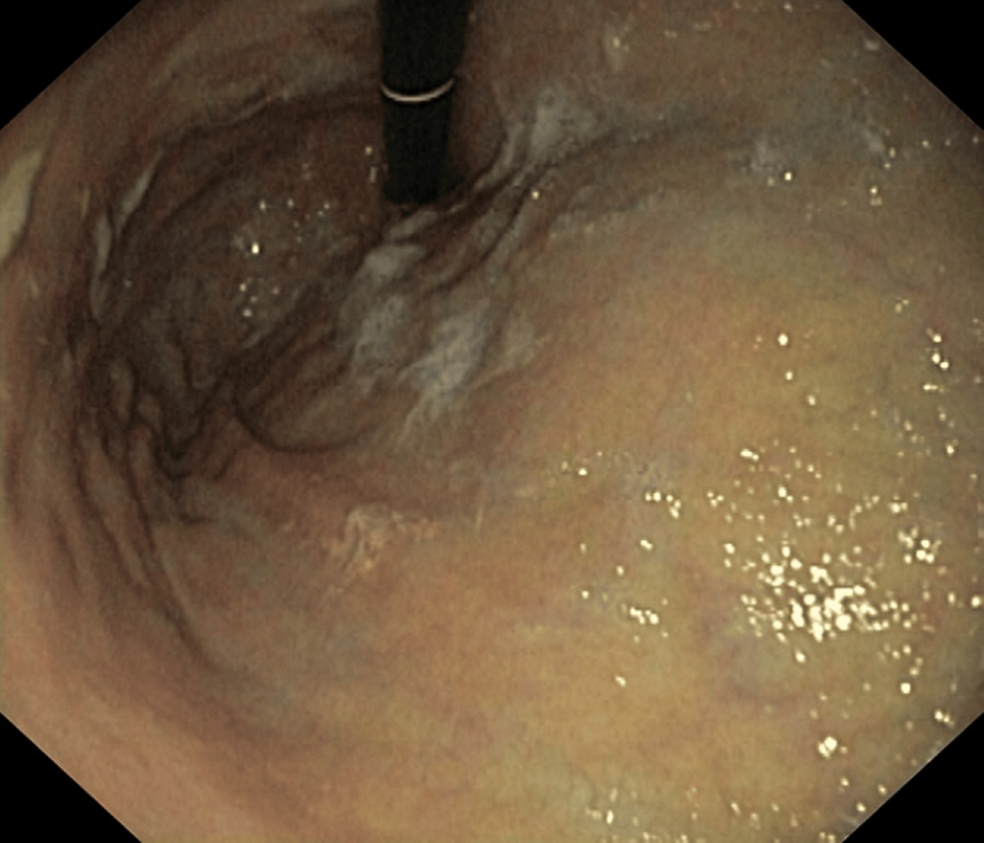
Fundic atrophy (fundus appearance)

The patient was a 56-year-old male with no specific medical history who sought medical attention for an episode of melena and stomachache. He also reported memory and loss of balance. Clinical examination revealed a cerebellar gait with impaired proprioceptive and vibratory sensitivity and a swollen and red right lower limb with a positive Homan sign. The blood count showed macrocytic anemia with a hemoglobin level of 11g/dl and an MCV of 130. Esophagogastroduodenoscopy revealed flattened fundic folds resembling a fundus appearance(Figure 1), and histopathological examination confirmed fundic atrophic gastritis with pseudopyloric metaplasia and lymphoplasmacytic infiltration.

Anti-intrinsic factor antibodies were positive, and anti-parietal cell antibodies were negative. Vitamin B12 levels were severely low at 42 pg/ml, while the vitamin B9 level was normal at 21.99 ng/ml. TSH and HbA1c levels were within normal ranges. An abdominal CT scan looking for a neoplastic syndrome showed no anomalies. Lower limb Doppler ultrasound confirmed the diagnosis of deep vein thrombosis (DVT), revealing extensive thrombophlebitis from the popliteal vein to the level of the deep femoral vein. The cardiac evaluation showed sinus bradycardia suggestive of secondary dysautonomia without major cardiac involvement, with an ejection fraction of 61%. Given this clinical picture, we diagnosed PA complicated by neurological and cardiovascular involvement. The patient was supplemented with vitamin B12 at a dose of 1000 ug/day for five days, followed by 1000 ug/week for a month, then 1000 ug/month, and received direct factor Xa inhibitor anticoagulant for DVT until vein permeabilization was achieved. Clinical and biological evolution was favorable, with a return to walking autonomy, disappearance of forgetfulness episodes, venous recanalization, and a control vitamin B12 level of 984 pg/ml.

## Discussion

Neurological manifestations of PA were first described in 1940, highlighting the crucial role of vitamin B12 in the isomerization of methylmalonic acid to succinic acid [3]. The classic neurological presentation of PA corresponds to combined degeneration of the spinal cord, involving both a posterior column syndrome and a pyramidal syndrome [4,5]. This presentation, sometimes only detected through electromyography, accounts for 20-30% of cobalamin deficiencies and PA with neurological involvement [4]. Other neuropsychiatric manifestations are highly varied, including sensory-motor neuropathies with a more or less abrupt onset and progressive worsening, facial paralysis, retrobulbar optic neuritis, cerebellar syndrome, and dementia, among others [4,5].

In our patient, there was a sensory-motor neuropathy and dementia. Several studies have examined the relationship between vitamin B12 deficiency and thromboembolic disease. Homocysteine, a metabolic precursor of vitamin B12, is recognized as a risk factor in atherothrombosis and is elevated in 95% of vitamin deficiency cases [6]. However, this complication is underreported in the literature and poorly represented in epidemiological studies. In the series by Loukili et al. (2004), only two out of 49 cases had a stroke, and one case developed femoral vein thrombosis [7]. Only a few cases in the literature have involved vascular involvement leading to the diagnosis of PA. Mesenteric vein thrombosis has been reported by Marie et al. [8], superior vena cava thrombosis and DVT of the lower limbs by Kharchafi et al. [9], and pulmonary embolism by Caldera et al. [10]. However, in the case reports of Marie et al. and Caldera et al., a coagulation abnormality was also associated with vitamin deficiency, contributing to the severity of the vascular event [8-10]. In our case, only vitamin B12 deficiency seems to explain the vascular event.

## Conclusions

Neurological and vascular complications are rare events in the context of PA. Nevertheless, healthcare professionals should remain vigilant about this possibility, especially when encountering patients with concurrent macrocytosis. This vigilance can help establish an appropriate diagnosis and implement proper management of this medical condition.
